# Data Collection Theory in Healthcare Research: The Minimum Dataset in Quantitative Studies

**DOI:** 10.3390/clinpract12060088

**Published:** 2022-10-26

**Authors:** Chun Shing Kwok, Elena-Andra Muntean, Christian D. Mallen, Josip Andelo Borovac

**Affiliations:** 1 Department of Post Qualifying Health Care Practice, Faculty of Health, Education and Life Sciences, Birmingham City University, Birmingham B5 5JU, UK; 2 Royal Stoke University Hospital, Stoke-on-Trent ST4 6QG, UK; 3 Smart Innovation Hub, Keele University, Stoke-on-Trent ST5 5BG, UK; 4 School of Medicine, Keele University, Stoke-on-Trent ST5 5BG, UK; 5 Clinic for Heart and Vascular Diseases, University Hospital of Split (KBC Split), 21000 Split, Croatia; 6 Department of Pathophysiology, University of Split School of Medicine (USSM), 21000 Split, Croatia

**Keywords:** routine data sources, routine collection of health information, design of data collection, information theory

## Abstract

There is considerable interest in data analytics because of its value in informing decisions in healthcare. Data variables can be derived from routinely collected records or from primary studies. The level of detail for individual variables in quantitative studies is often disregarded. In this work, we aim to present the concept of a minimum dataset for any variable. The most basic level of data collection is the value of a variable. In addition, there may be an indicator of severity and a measure of duration or how long the value has been present. The time course defines how the values for a variable fluctuated over time. The validity or accuracy of the values for a variable is also important to avoid spurious findings. Finally, there may be additional modifiers which drastically change the impact of a variable. In conclusion, the minimum dataset is a framework which can be used for the purposes of study design and appraisal of studies. Not all data requires full consideration of the minimum dataset framework for each variable, but the framework may be important if more detailed results are desired.

## 1. Introduction

### 1.1. Data Analysis and Research

There is considerable interest in data analytics because of its value in informing decisions in healthcare. Data is collected from routine health records or prospectively acquired for the desired purpose. Statistical analysis is then performed on the acquired data to generate results for interpretation. Potential areas for concerns in data collection and quantitative analysis are related to the need to control or adjust for variables to limit any biases in the results. Results where there are limited biases are important because clinical decisions are often made based on these results. Decision making based on the outputs of the analysis of data has advantages over decisions made solely on anecdotal observations or inferences.

Research is increasingly competitive and although there is scientific scrutiny within the academic cycle from grant funding, ethics and peer review, there is an ever-increasing number of platforms for publishing, including traditional print publications, blogs and social media. The COVID-19 pandemic accelerated the growth of preprint platforms as a rapid way of disseminating biomedical knowledge [1], but on the other hand, it strongly stressed the need for high quality, independently evaluated and robust research. A careful assessment of the quality of the data used in healthcare research should be a key component of the independent review process. The assessment should also consider the process of data collection, which is often neglected but it could be more important than both the data and the analysis. Quality of data collection strongly contributes to quality and trustworthiness of the results, as poor-quality data entry often leads to unreliable data output—this is also known as the “garbage in–garbage out” concept [2]. This problem can burden individual studies and trials but can also contaminate publications that aggregate data from multiple studies, such as meta-analyses [3].

The objective of this work is to present the concept of a minimum dataset for any variable which can be used for the purposes of study design and appraisal of quantitative studies.

### 1.2. Routine Data in Healthcare Settings

In healthcare settings, data used for research can be derived from routinely collected records of clinical practice or from evaluations such as clinical research. When using routinely collected data for other purposes, it is essential to understand the purpose and motivation behind its original collection. Data may be used for billing purposes so that healthcare organizations can be reimbursed either by the individual in private healthcare settings or the government in countries where there is public healthcare. The data is also a clinical record which acts as a medicolegal document which enables some accountability that the patient had received a diagnosis and the associated management. The data as a clinical record has addition importance to inform others about the patient care received and ongoing care advice when the patient is discharged. The extent of data collected, and the completeness of the collected data, can be questionable. There is no guarantee that all data of interest to a specific question is present. For accountability, routine records can be evaluated in a service evaluation or a clinical audit to ensure that expected practices and standards are met.

### 1.3. Clinical Research and Data

Studies in clinical settings where the researchers actively collect data for a pre-specified research question is usually superior in terms of data quality. Data in observational studies are mainly not collected for pre-specified purposes, there is lack of blinding and inadequate control for unmeasured confounders [4]. The reliability of observational studies is burdened by substantial variability and largely depends on the design of a particular registry/local database, methods used for the analysis and the quality of the data that is recorded [5]. An example of study designs that are at the top of the evidence pyramid are the systematic review, meta-analysis and randomized controlled trials [6]. Active data collection enables the collation of data that is relevant to the question posed in randomized trials. Data is collected to enable the comparison of baseline characteristics between the groups that receive different treatment to improve the likelihood that any effect observed is due to differences in treatment allocation. Although trials have great value in answering many research questions, there are some questions which cannot be answered with trials, including questions relating to disease incidence and prevalence in a population and observations of clinical practice and associated outcomes. This is probably best illustrated with an example.

Suppose one wants to study a population of patients who are confirmed by a primary care setting to have atrial fibrillation (AF). This group of patients may be of interest to a researcher who wants to plan a study of AF or someone who wants to evaluate what happens in terms of clinical care and outcomes for this group of patients. As most general practices have electronic records, a relatively straightforward approach would be to search these primary care records to identify people with an AF diagnosis. This type of search, if properly conducted, will identify all patients in the practice with a diagnosis of AF who have been coded correctly in the records. However, this only identifies those with clinically known AF and those who do not have symptoms or have not undergone electrocardiographic testing would not be captured. To reduce the risk of missing the silent cases of AF, an electrocardiogram would have to be performed on all patients in the practice in order to ensure that all cases are at least screened with an electrocardiogram for AF. This is important for a research study that aims to randomize patients with AF for different treatments and has less emphasis on ensuring that all cases are captured, compared to a study of the burden of AF among patients in the community.

### 1.4. Challenges in Data Collection in Research

The challenges of new data collection are that it is expensive and time consuming. In order to prospectively collect data for clinical research approvals from institutional review boards, governing bodies and ethical committees must be sought. In addition, there is the cost of conducting the research, time for the study to take place and the need for research grants, which are needed to secure the cost of the salary for the staff involved in data collection, data storage, any compensation for those involved in the research and operating costs for institutions where the research is taking place. This potentially creates barriers to data collection as protocols need to be prepared, assessed, revised, agreed upon and checked that they are in accordance with guidelines for research practices. For some research questions, new high-quality data may be required to derive meaningful results, but this may be expensive and labor-intensive work. To increase the quality of observational data, various quality control procedures and guidelines for data acquisition, quality and curation have been developed in various healthcare settings [7,8,9].

### 1.5. Model of Data Collection in Healthcare Research

The current model of research typically involves an academic who applies for funding and guides the applications through all the regulatory bodies. This academic and the research team will oversee the project and be responsible for its scientific integrity. However, the physical collection of data is often carried out by other research personnel who are employed and have the responsibility to follow the defined protocol, and other members who may be involved in the analysis and presentation of the results.

The aim of data collection in research should be to capture data as accurately as possible and to minimize the chance of bias. To determine what is going on requires critical thinking of the data that is collected and how it is handled. Data collection should be informed by evidence rather than by opinion or anecdote. There are many important questions:

Who determined what is collected?

Does the research team have accurate knowledge about the condition that is studied? Familiarity with the subject in terms of pathophysiology is needed in order to reduce the chance of omitting the collection of important variables.

A standardized approach is important to reduce the risk of bias related to familiarity.

Does that researcher who designed the study and methodology understand what happens in clinical practice regarding that condition? For example, if one is studying a cardiology condition, it is easy to consider the first point of contact with a cardiologist as the starting point, but many other healthcare professionals may have been involved with the patients care such as general practitioner, community healthcare professional, paramedic and emergency department doctor. The person determining what is collected should also know about practices outside of the center they work at as there may be differences in care comparing rural and urban settings.

Does the researcher who designed the study and methodology understand what has been done in studies of a similar nature that have been done previously? It is helpful to conduct a systematic review to at least understand what has been done previously. Scrutiny of existing studies enables the new work that is taking place to have at least the same or better standards in methodology as the previous works in the area and any limitations in research methods that have been identified could be addressed for the future studies.

Is the proposed data to be collected reliable? The data collected should be accurate and internally valid. The ideal data is that which also has external validity and makes the findings generalizable in most settings.

Does that data collector understand how to collect data in a way that can be easily analyzable? Ideally, data collectors should be familiar with analytical approaches of the research question so that the desired accuracy of the variable will be recorded. For example, a primary study of patients with hypertension as an outcome may require a mean value for blood pressure for participants. In contrast, a study which utilizes hypertension as a covariate in a population may only require that hypertension is more simply coded as “yes” or “no”.

Academic researchers who lead studies often review the literature in order to determine how research data should be collected. This approach may make the methods more reliable in the context of the existing literature. However, there may be flaws in the methodology of published research as it could just perpetuate the same problems. Furthermore, what may work for one setting may not be successful when the same methods are applied in other settings. For example, an expert from a specialist center defines data to be collected, including patient outcomes, based on his personal experience and understanding of the literature. However, what might be underappreciated in the defined evaluation is that the collected data may not capture factors such as those which take place outside of the hospital or within the hospital which they are not familiar with, such as the care from the emergency departments or other specialties which may impact patient outcomes. Furthermore, there may be patient-specific factors that influence their care which may not be captured. An example of this may be the patient decisions not to take medications or personal wishes such as desire not to be admitted as an inpatient. These factors are particularly important when studies find results that are contrary to expectations. Rather than dismissing the data as incorrect, there should be a critical assessment and consideration about whether improved data collection methods may result in an understanding of why the findings were found.

In order to support better quality data collection, the following framework called the minimum dataset is proposed.

## 2. The Minimum Dataset

### 2.1. Basic Unit of Data

**Figure 1:**
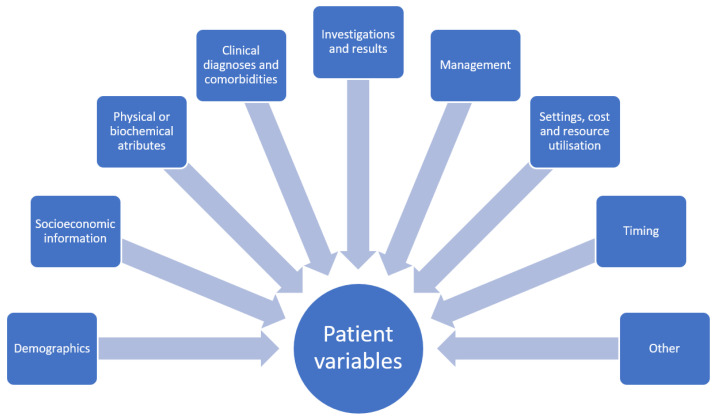
Broad categories of dataset variables.

A dataset for quantitative study contains variables. Each variable will have a name and there will also be assigned possible values for the variable. Depending on the variable type, the values may be continuous such as age, or categorical such as sex which may be male or female. Variables may also be ordinal where there is rank associated with the categories. In addition, there are variables with values that are narrative which contain descriptive text and these variables may not be easily utilized in statistical analysis without processing because the text may require conversion into categories before analysis can take place. In healthcare research, these variables could be a patient physical descriptor such as age, height, or body mass index as well as other defining characteristics including location of residence, occupation, or income. Variables could also depict symptoms such as chest pain or shortness of breath or management received such as receipt of chest X-ray or echocardiography and place of care (i.e., general practice, emergency department, hospital inpatient setting, etc.). The treatment provided, such as antiplatelet medications or antihypertensive medications and associated responses, are other variables potentially of interest. There may also be more abstract concepts as variables such as reasons why the patient did not take medications or visit a doctor. In general, the domains where variables fall under in clinical research include demographics and socioeconomic information, physical and biochemical attributes, clinical diagnoses, investigations and results, management and place of management, settings, cost and resource utilization, timing, and other variables such as explanation (why patient refuse treatment, why they did not present to doctor, etc., see Figure 1). The classification of these variables is based on statistical theory, but what is not considered is whether the variables are evaluated for the necessary detail for analysis and research questions.

A fundamental part of a study is defining the variable of interest that is relevant to the research question. Although with some clinical knowledge it is possible to determine potential variables relevant for a research question, there may be need for collection of some additional data to determine what is relevant as well as conducting a review of what previous studies have done.

**Figure 2:**
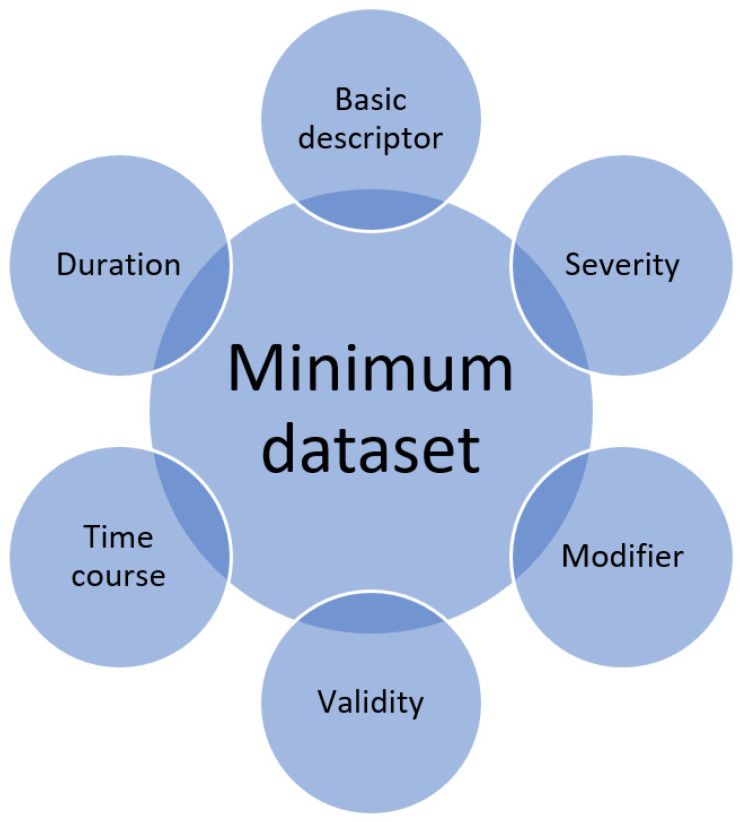
Minimum dataset for a single variable.

The important considerations for each variable are defined as follows in Figure 2.

### 2.2. Basic Variable Description

The most basic level of data collection is the value of a variable. For continuous variables, this would be the exact value similar to that for age (e.g., 65 years old). For categorical variables, it would be a category such as smoking status, i.e., classified as current smoker, ex-smoker or non-smoker. Some variables, such as the examples given above, do not require further consideration of detail within variables, however, for the best data collection, other factors may need to be considered.

### 2.3. Severity

In addition, regarding the presence or absence of a value, there may also be some indicator of severity. For example, a patient may have shortness of breath in the context of chronic heart failure. The New York Heart Association classification [10] is used to determine the stage of heart failure from class I where there are no symptoms and no limitations to ordinary physical activity to class IV where the patient is unable to carry out any physical activity without discomfort and there are symptoms at rest. An alternative grading system for heart failure may be the consideration of the left ventricular ejection fraction which is represented as a percentage; a value of less than 40% represents reduced ejection fraction whereas an ejection fraction of greater than or equal to 50% represents a preserved ejection fraction [11]. Similarly, biochemical measurement of natriuretic peptides from the blood sample of a patient with heart failure might also indicate disease severity [12]. The severity marker may be agreed upon similar to the examples described or decided by the researchers with some justification where there is no acknowledgement system of evaluating severity. The optimal choice of severity indicators for a variable depends on what options are available and the exact research question.

### 2.4. Duration

There is a duration from the first onset of a value for a variable until the time of evaluation. Understanding the duration of the value for a variable may have clinical and research implications. For example, a variable may be the presence or absence of coronary artery bypass graft (CABG) surgery. There is a significant difference between a patient that had the procedure in the last few months compared to a patient who had the procedure 20 years ago. The patient who had a recent the procedure would have benefited from current evidence-based treatments and the types of complications they may have will be different from someone who had it many years ago. A patient who had recent CABG surgery will be at risk for infection and bleeding and early post-procedure complications might also include arrhythmias. On the other hand, CABG procedures that were done many years ago that were without complications may be at risk of blockage of the grafts and angina and acute coronary syndrome. It is important to consider that the time of detection of a value for a variable may not actually be the time of onset. For a condition to be diagnosed, a patient must present to a healthcare professional who can make the diagnosis. This is important for congenital conditions where patients are born with a problem such as a bicuspid aortic valve. The variable may be revised with the onset of symptoms with their abnormal valve rather than duration of the disease. Duration can also have implications on management such as the case of AF. If patients present within 48 h of onset, the likelihood of clot formation is lower, so they may be amenable to electrical cardioversion without a duration of anticoagulation to stop clots from forming or progressing. The important consideration about the onset of variable in relation to its duration relates to how reliable the variable reporting is. A patient may say they have had palpitations in the last 24 h before presentation and detection of AF, but they may have had the condition for longer but were only aware of it in the last 24 h.

### 2.5. Time Course

Time course is a complex variable which often requires in-depth disease understanding to appreciate. The concept of the time course can be considered with different levels of detail. At the highest level of detail, there may be detailed documentation of specifically how the condition changed over time in relation to severity. It is also possible to consider the variable in less detail but more from a general term in the sense that it has stayed the same, became worse or became better. This process compares the initial state to the present state. The trajectory of severity may matter over time as the reviewer suggests which is better captured with the detailed data than the more general approach of the initial versus the final state. The premise of the minimum dataset is that the time course should be considered but whether it is with high detail or less detail is dependent on how the variable fluctuates over time, which depends on the variable itself.

**Figure 3:**
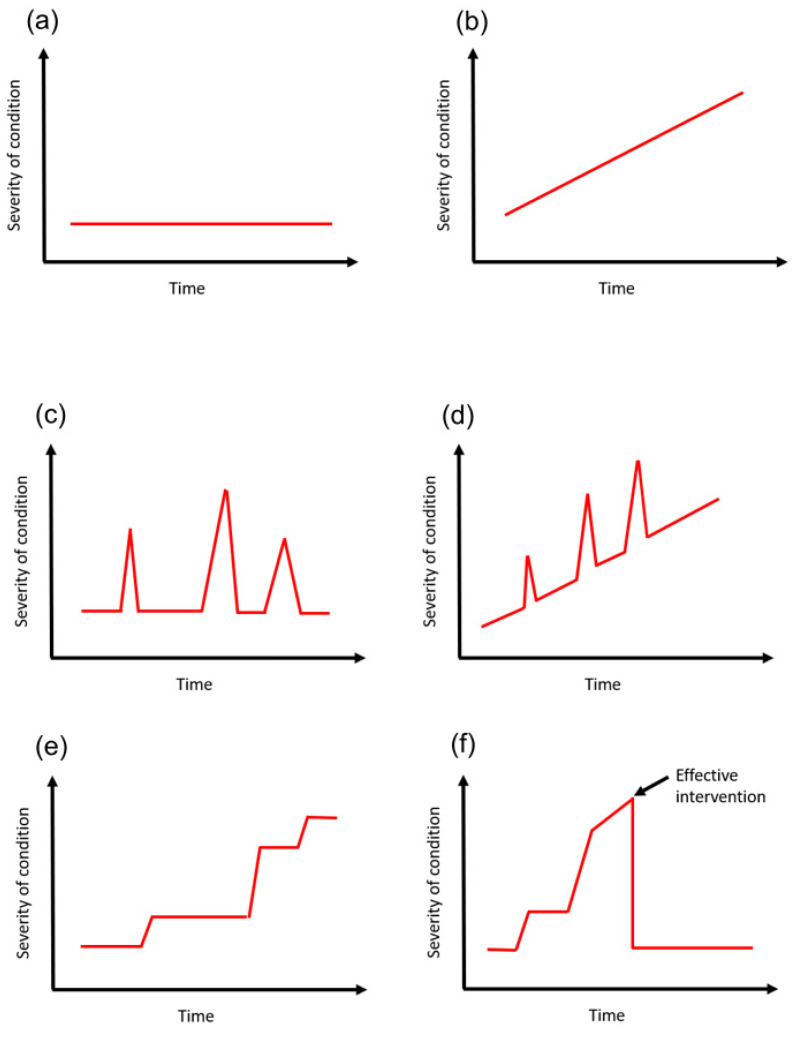
Temporal trajectories of variables over time. A condition can be stable with no changes on the *y*-axis over time (**a**). It can also progressively decline (**b**). Between both models, you can have acute deterioration and recovery (**c**,**d**). You can also have stepwise progression where there are deteriorations with decline over time (**e**). There are also other models where modifiers can change the time course such as surgery to alleviate the problems (**f**).

An example may be a patient with type 2 diabetes mellitus who had been diagnosed 20 years ago. The course of the condition can be variable. A patient who does not control risk factors for disease progression such as blood glucose levels may end up with complications such diabetic nephropathy, retinopathy, neuropathy, and cardiovascular disease such as (ischemic heart disease, stroke, and peripheral vascular disease) and require oral hypoglycemics, insulin, renal replacement therapy and other procedures. This is very different from a patient who was diagnosed 20 years ago but made major changes to lifestyle including weight loss, increase exercise, smoking cessation and dietary changes such that they remain diet-controlled over the 20 years. The time course can be best illustrated as a graph with some marker of severity on the *y*-axis and time represented on the *x*-axis (Figure 3).

### 2.6. Validity

What is often overlooked is how valid a variable is during data collection. Validity is defined as the extent to which a concept is accurately measured [13]. There are different ways of collecting data which has variable levels of validity. A simple way of collecting data is from medical records. The validity of the data depends on the clinical teams first identifying the variable and secondly documenting the variable on medical records. Even if the variable is identified during the course of the patient journey, there is no guarantee that it merits documentation and coding in the clinical record. For example, contrast-induced acute kidney injury is a relatively common complication after coronary artery angiography and percutaneous coronary intervention [14]. The discharge records may only feature the pertinent details for the heart attack and omit the renal impairment. This may be the case where there was mild dysfunction or recovery of the renal function during the inpatient stay. Even if the clinical team documented the problem in the clinical notes, there is no assurance that the records which were reviewed by the researcher were consistent with the location where the renal failure was documented. Medical records are complex as different clinicians such as doctors, nurses and other healthcare professions record entries in different places and often use different systems. What is documented in written notes may not be captured in electronic patient records and vice versa. Frequently, there are assumptions that a review of medical records is satisfactory but there is no clarity regarding the extent to which the records were reviewed. Electronic records may have been reviewed for example but not the paper records. The researcher needs to know exactly how data is collected which may vary considerably at different sites. Some places will have purely electronic records whereas others may have mixed electronic and paper records. Even if the information is electronic, there is no guarantee that has been inputted in the proper location. In addition, there may be multiple independent records, which is often the case for historical data where organizations underwent a transition from paper records to electronic records over time.

The second approach is to collect data directly from the patient. Some variables, such as a blood test values, are more reliable compared to asking a patient to recall whether they had existing health problems or abnormal blood results. There are two important considerations here. For example, a question in data collection may be to ask a patient whether or not a he or she has hypertension. This question relies on the patient to know what conditions he or she has but also that he or she was previously evaluated for hypertension. The question may be satisfactory for patients who are known to have undergone testing for hypertension and been told of the result. However, this may be a problem for patients who have not been tested. The most valid way of testing for a variable is where you actively look for the variable and you exclude it based on testing or physical evaluation (like checking a blood pressure). This approach avoids problems associated with patients having silent problems that were undetected.

Validity also has importance for data collection in relation to outcomes for studies. For instance, the COVID-19 vaccine is used to reduce the risk of COVID-19 infection but there are concerns that it may increase blood clots [15]. If one wanted to know whether COVID-19 increases the risk of pulmonary embolism (PE), one way would be take all the patients with the vaccine and determine from hospital records the rate of PE admissions. However, this method relies on patients having symptoms of PE and presenting to a hospital and the event being diagnosed accurately and recorded. If patients suddenly die and the blood clot may only be found on autopsy may or may not be captured through hospital data. The same applies for asymptomatic PE, where patients do not present for clinical care. Not that it is practical to do, but a more robust way to determine the significance of PE is testing every patient for PE who has had a COVID-19 vaccine. This may not be practical because of the cost of doing a CT pulmonary angiogram for every patient and the associated radiation and contrast risk. Although it will not collect all cases of PE, one approach may be to change the question from the rate of PE associated with COVID-19 vaccination to clinically relevant PE, where it considers PE cases which significantly impact patients and seek healthcare attention.

### 2.7. Modifiers

Modifiers are variables which impact other variables. They are specific additional considerations that are necessary for some variables. The presence of a modifier is determined by clinical understanding of the natural history of a condition. Therefore, pure statistical exercises without clinical input may fail to capture the significance of modifiers. For example, a person may have had a diagnosis of skin cancer. This may have been treated with an operation to remove the skin lesion and the operation is a modifier of impact of the skin cancer. Consideration of the modifier is important as successful clearance of the malignant skin lesion may render the patient effectively cured without a major impact on any long-term consequences from the initial cancer (they will still have risk factors and may develop another cancer). However, the history of skin cancer in a patient may be important in some research questions as a patient may have received chemotherapy or radiotherapy which may have long term consequences. Modifiers require some clinical insight to determine if they need to be considered for each variable and how they should be best handled.

## 3. Discussion and Implications

The purpose of this framework is not to say that all data requires this in-depth consideration. Most variables do not require considering every element highlighted in the framework. The purpose is to provide an outline to consider if one desires a comprehensive data set.

The proposal of the minimum dataset calls for more detailed thinking about variables and data collection in quantitative research. Contemporary research has come to a state where many journals focus on what studies find and how they impact practice as opposed to whether the data collection is of sufficient quality or requires improvement. It is assumed by most researchers that what they propose to collect or the data that a research reviewer are presented with is satisfactory without always considering the level of detail of the information collected. This work calls on researchers and reviewers to think more critically about the variables in datasets.

Quantitative observational research includes descriptions of variables that are collected. Whether each individual variable is collected too superficially or has the sufficient detail for full understanding of its significance is not often considered. Awareness of the minimum dataset calls on a researcher when designing the methodology of a study to carefully think about each individual variable and how they may want to collect more detailed information if appropriate. The same awareness is important for peer reviewers of manuscripts as variables may be presented but some of these may not have sufficient detail that is relevant for the research question. Therefore, the concept of the minimum dataset raises the question of whether a variable is captured to the necessary detail for the question and the proposed areas to consider for any given variable are the basic descriptor, duration, time course, validity, modifiers and severity.

A consideration of data validity and reliability is the “hardness” or “softness” of the variable. Hard variables are those which are nearly unequivocal; there are exceptions such as incorrect documentation. Variables that can be hard include mortality, age, blood test results and physical measurements, such as weight and height if taken properly on calibrated equipment [16]. Although errors do occur in measurements, assuming that the assessor is trained properly, the errors that occur may be related to inherent calibrations or failures in the equipment. Soft variables are those that are detected and there may be a degree of ambiguity and interpretation. This could be clinical signs where there is no standardization of eliciting features. For example, the detection of an elevated jugular venous pressure may be very different for a junior doctor with little experience compared to a heart failure specialist consultant. Hard variables are more reliable whereas soft variables may benefit from the use of surrogate measures, such as invasive central venous pressure measurements or correlation with peripheral oedema and lung base crepitations to corroborate an estimate of jugular venous pressure. In a similar example, surrogate endpoints or outcomes guiding clinical practice in treatment of arterial hypertension include left ventricular hypertrophy, microalbuminuria, arterial stiffness and carotid intima-media thickness [17]. Similarly, quality of life measures in which patients describe and grade the impact of the disease and/or treatment for their disease on their life is also considered “soft” and subjective data [17]. The softness or hardness of a variable adds to consideration of the attributes of data that is often overlooked. Soft variables are those that we can accept that there may be a larger error in value, whereas hard variables are those that we expect to be correct. The reality is that most variables are on a spectrum with a varying degree from hard to soft. In modern clinical trials, the robustness of findings might be inspected by performing sensitivity analyses that can reveal if obtained results are affected by changes in methods, models, values of unmeasured variables or assumptions [18].

There is also a palpable difference in perceiving the importance of endpoints in research from a patient versus physician perspective. Endpoints that are important for clinicians might not be that important for the patient and vice-versa. Therefore, patient-reported outcomes should be integrated in clinical trial endpoints, such as in the oncology field [19].

Appreciating data and its completeness is an important part of the critical thinking process when reviewing literature. It is important that the methodology of a paper be given full consideration when interpreting the findings. A good transparency test when reviewing a study is considering whether one can reproduce the study given unlimited resources. Some authors argue that word count limits in scientific publications preclude making detailed descriptions of their data collection methods. However, most journals do accept supplementary data and the protocols can also be published on institutional webpages. To understand why events happen, there are more factors than just those that are clinically relevant. For instance, how the healthcare system is delivered, and how data is recorded in the setting where the research took place may be important. In a private healthcare setting, patients may not be able to afford treatment. In this case, patients may experience poor outcomes because of other factors rather than failure to offer optimal care. On the other hand, in a public healthcare model, not all treatments may be available because of cost, so patients may have poor outcomes because the healthcare providers lack the resources to provide the treatment.

The proposed minimum dataset may have value in reviewing protocols, study planning and peer reviews of a manuscript. When reviewing study protocols and peer reviewing a manuscript, the authors of the protocol or manuscript will define what data that should be collected, which typically includes multiple variables and specifies the different possible values for each variable. Appraisal of the protocol or manuscript with the minimum dataset enables systematic consideration of whether each variable may need more detail, considering some of the elements of the minimum dataset. This has relevance when the findings of a study are contrary to what is expected. In such cases, there should be some explanation and it may be related to the need to have more detailed understanding of the variable to better estimate the true results. For example, a variable could be the presence or absence of diabetes mellitus. A study may find that diabetes mellitus does not increase mortality compared to the population without diabetes. However, it is well known that diabetes increases risk of cardiovascular disease and mortality [20]. No difference in mortality may relate to the cohort studied being low risk without severe or end-stage diabetes so that capturing the data on duration and severity of diabetes mellitus might help elucidate why no increase in mortality was detected.

Despite the framework proposed, some questions remain about data collection. There is no best way to determine what is needed to be collected. Reviewing the literature can be helpful but critical thinking is still required. Judgments by researchers need to be made regarding the depth or detail for the data that needs to be collected. It is unclear how researchers can be sure that they have not missed any important data that could influence their findings. A group of researchers with clinical and methodological expertise can sometimes be helpful. Clinical expertise and understanding patient actions and care practices should also have value in decisions about data collection. For example, the understanding of the time course may not be clear. This may be the case for a variable such as a previous COVID-19 infection and the long-term consequences. In this case, it may be necessary to collect the data and define what courses there may be. Even for another example, such as the long-term outcomes after having an acute coronary syndrome, consideration of data from more than a decade ago may not reflect the prognosis for contemporary practice because of all the improvement in care. There are also important subjective judgements that may need to be made. For example, for a severity of a condition, it may be a three-tier classification of mild, moderate or severe. However, it could also be a five- or greater tiered scale. Decisions about how to stratify severity into different groups likely requires some justification where there are not well-acknowledged cut-offs. The reality is that the data required to answer the question is likely to be question-specific.

The challenge of collecting data is determining how much data is needed. Theoretically, there are limitless data that could be collected but it is best handled for analytic purposes, and data variables should be kept to a minimum needed to answer the research question. Statistically, most analyses are interested in principal components or variables which are mutually exclusive (i.e., each variable are independent and changes in one do not affect the others). However, variables in real life have a degree of overlap as the patient who is obese is also more likely to be sedentary, have high cholesterol, high blood pressure, existing cardiovascular disease and conditions such as obstructive sleep apnea and hypothyroidism. If the outcome of interest is death, many of these factors will be associated with increased risk of mortality, and considering some of these variables may have altered the association with death depends on whether other variables are included in the model.

Scientific publications have a section in the discussion devoted to limitations that are recommended by most checklists for reporting studies such as the CONSORT statement [21] and STROBE statement [22]. In general, the consideration of limitations related to the collected data is frequently ignored or discussed superficially.

The consideration of the minimum dataset has limitations. First, this approach may complicate data collection and analyses, making it more time consuming. It may be argued by some researchers that collecting the variable on a basic level may be satisfactory and an example of this may be the presence or absence of diabetes mellitus. However, the assumption in this case is that the duration, severity and time course of the diabetes mellitus does not matter. Ideally, the assumption must be proven that these additional levels of consideration for the variable diabetes mellitus are important for the research question of interest. This may make data collection and analysis more challenging. In addition, these additional levels may be a source of variability. This variability may make underpowered studies less likely to show statistical significance between study groups because the effect of introducing the additional elements of the minimum dataset is like adding additional variables to a model. Secondly, the practical application of the minimum dataset mainly applies to the planning of data collection and defining the variables to collect, and there are cases such as routine data where the data is already collected. In these cases where the data is collected, what you have is what is collected, and it may not be possible to add additional detail that is not available.

## 4. Conclusions

In conclusion, we propose a framework called the minimum dataset which could be considered in the design of studies that involved data collection or in the appraisal of studies that collect data. This framework can be used to more comprehensively capture variables beyond simply considering variables a basic descriptor alone. The potential importance of this approach is that more detailed data collection may be achieved for each variable so that the understanding of the associations between variables and outcomes can be improved. Future work is needed to determine if this approach can improve data collection to produce higher quality datasets and results.
